# Translational Control of Arabidopsis Meristem Stability and Organogenesis by the Eukaryotic Translation Factor eIF3h

**DOI:** 10.1371/journal.pone.0095396

**Published:** 2014-04-15

**Authors:** Fujun Zhou, Bijoyita Roy, John R. Dunlap, Ramya Enganti, Albrecht G. von Arnim

**Affiliations:** 1 Genome Science and Technology Program, The University of Tennessee, Knoxville, Tennessee, United States of America; 2 Department of Biochemistry, Cellular and Molecular Biology, The University of Tennessee, Knoxville, Tennessee, United States of America; 3 Division of Biology, The University of Tennessee, Knoxville, Tennessee, United States of America; The University of Tokyo, Japan

## Abstract

Essentially all aboveground plant tissues develop from the stem cells in the primary shoot apical meristem. Proliferation of the stem cell population in the Arabidopsis shoot apical meristem is tightly controlled by a feedback loop formed primarily by the homeodomain transcription factor WUSCHEL (WUS) and the CLAVATA ligand-receptor system. In this study, it is shown that mutation of a translation initiation factor, eIF3h, causes a tendency to develop a strikingly enlarged shoot apical meristem with elevated and ectopic expression of *WUS* and *CLAVATA3 (CLV3)*. Many of the mRNAs that function in apical meristem maintenance possess upstream open reading frames (uORFs), translational attenuators that render translation partially dependent on eIF3h. Specifically, the mRNA for the receptor kinase, *CLV1*, is undertranslated in the *eif3h* mutant as shown by transient and transgenic expression assays. Concordant phenotypic observations include defects in organ polarity and in translation of another uORF-containing mRNA, *ASYMMETRIC LEAVES 1* (*AS1*), in *eif3h*. In summary, the expression of developmental regulatory mRNAs is attenuated by uORFs, and this attenuation is balanced in part by the translation initiation factor, eIF3h. Thus, translational control plays a key role in *Arabidopsis* stem cell regulation and organogenesis.

## Introduction

In eukaryotic cells, gene expression is highly regulated, often at multiple levels, such as transcription, mRNA structure and stability, translational control, and protein degradation. Translational regulation is arguably least well characterized, and questions concerning the mechanism of translational control abound. In plants, translation is regulated by small metabolites as well as environmental conditions (reviewed in [1]–[3]). In contrast, how translational regulation contributes to plant development remains largely uncharted territory. Mutations that affect specific proteins of the large and small ribosomal subunits, which were recently discovered in genetic interaction screens, suggest a role for translational control in leaf polarity [4]–[7]. Moreover, mutations in *RPL24B/SHORTVALVE (STV*) cause defects in organ initiation, vascular patterning, and gynoecium structure that could be attributed to undertranslation of mRNAs for transcription factors of the auxin response factor (ARF) class [8].

Among the eukaryotic translation initiation factors, eIF3 is by far the most complex, consisting of 12 subunits in Arabidopsis [9]. eIF3 participates in almost all major steps during initiation, such as tRNA charging of the 40S ribosomal subunit, loading of the charged 40S onto the mRNA’s 5′ Untranslated Region (UTR), mRNA scanning and start codon recognition, and translation reinitiation (reviewed by [10]). The functions of the individual eIF3 subunits remain to be fully characterized. The h subunit of eIF3 is not conserved in budding yeast, but forms part of the functional core of mammalian eIF3 [11], [12]. In Arabidopsis, carboxyl-terminal truncation alleles of *eIF3h* cause under-translation of specific mRNAs, many of which harbor multiple upstream open reading frames (uORFs) in their 5′ leader [13], [14]. uORFs generally inhibit translation because a ribosome that has translated the uORF must terminate translation, resume scanning and acquire fresh translation initiation factors before it can translate the main ORF downstream. eIF3h ameliorates the inhibitory effect of specific uORFs in part by promoting the reinitiation competence of the translating ribosome [15]. The *eif3h* mutant shows auxin related phenotypes such as pin-formed inflorescence shoots, misexpression of auxin related genes, and poor translation of ARFs [14], [16], [17]. However, the *eif3h* mutant displays additional pleiotropic developmental phenotypes, such as growth retardation or growth arrest. It has remained unclear how under-translation of specific mRNAs causes these macroscopic phenotypes.

The plant tissues above ground ultimately develop from the stem cells in the shoot apical meristem (SAM). In Arabidopsis, the stem cell population in the SAM is tightly regulated by the CLAVATA-WUSCHEL (CLV-WUS) circuit (reviewed in [18]). CLV3, an extracellular peptide produced in the outer cell layers in the central zone of the SAM, is the ligand for the receptor kinase CLV1 [19]–[21]. In response to the CLV3 signal, CLV1, the related receptor-like kinase RPK2/TOADSTOOL, and the heterodimer of CLV2 and CORYNE, restrict the spatial expression of the homeodomain transcription factor, WUSCHEL (WUS), to a small cohort of internal cells that form the organizing center of the SAM. Besides other target genes, WUS induces the expression of *CLV3*, whereby a negative regulatory feedback loop is formed to ensure the stability of the stem cell population [19], [22]–[27].

Here we describe that a mutation in eIF3h causes a variety of defects in SAM maintenance that range from subtle defects in organ positioning and organ polarity to a massively enlarged, yet eventually quiescent, SAM. Translation assays revealed that eIF3h supports the efficient translation of the mRNAs for *CLV1* and the leaf transcription factor, *AS1*, which contain uORFs in their 5′ UTR. Mistranslation of these and other mRNAs in the *eif3h* mutant may disrupt the otherwise robust feedback circuits that underlie SAM maintenance and organ specification. Thus, the *eif3h* mutation amounts to a genetic perturbation that unveils a role for translational control in Arabidopsis SAM function and organogenesis.

## Results

### The *eif3h* Mutant Plants have Growth Defects in the SAM

**Figure 1:**
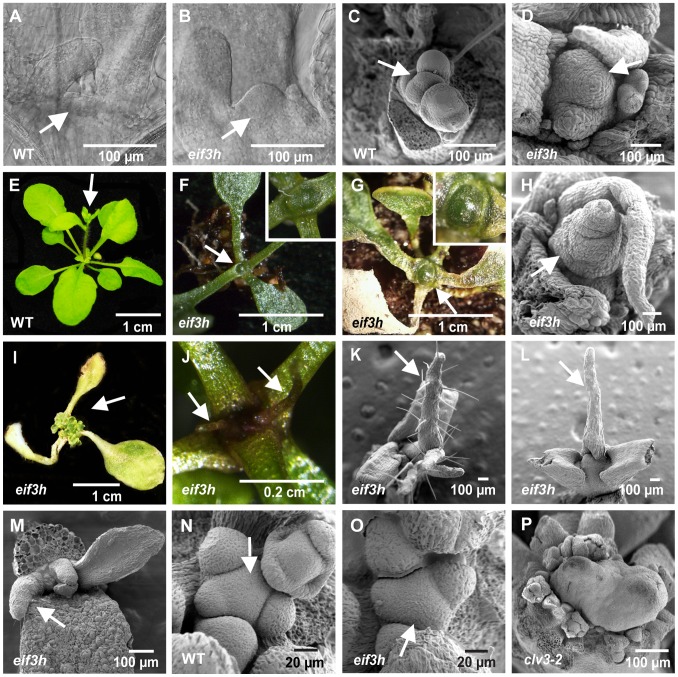
Defects of *eif3h* mutant Arabidopsis in shoot apical meristem maintenance. (**A–B**) 12 day old SAM imaged by differential interference contrast after clearing with chloral hydrate; arrows point to SAM. (**A**) wild type. (**B**) *eif3h* mutant. (**C–D, H**) Scanning electron micrographs of 3 week old wild-type inflorescence meristem (**C**) and equivalent in *eif3h* (**D and H**). Note enlargement of cells and of the entire SAM in *eif3h*. Arrow points to the SAM. The meristem in (**D**) is fasciated, i.e. branched into two, and both (**D**) and (**H**) have formed a radialized leaf. (**E–G**) Enlarged shoot apex in *eif3h*. White arrows point to the inflorescence (in wild type) or the shoot apex (in *eif3h*). (**E**) Wild type. (**F**) The enlarged quiescent *eif3h* SAM (inset shows a close-up of the apex). (**G**) Further enlarged dome-shaped *eif3h* SAM prior to senescence (inset shows a close-up). (**I**) Reactivated *eif3h* SAM with multiple apices initiating. (**J–M**) Filamentous organs emerging from the *eif3h* apex (arrows). (**J**) 3 week old *eif3h* mutant. (**K**) Filamentous organ with trichomes on 3 week old *eif3h* apex. (**L**) Filamentous organ without trichomes on 3 week old *eif3h* apex. (**M**) A filamentous leaf on a 1 week old *eif3h* apex. (**N–P**) Inflorescence apices. Arrows point to the SAM. (**N**) Wild type. (**O**) *eif3h*. (**P**) 3-week old *clv3-2*.

Unlike wild-type Arabidopsis plants, which always initiate a functional inflorescence from the shoot apex under normal growth conditions, a large proportion of *eif3h* mutant plants never initiated an inflorescence (33%, 45 out of 135). Closer inspection revealed growth defects in the shoot apex. Meristem enlargement could be seen as early as twelve days after germination (**Figure 1A, B**; **Table 1**) but was not reliably detected before that time. At twelve days, the *eif3h* mutant meristem had a slightly larger diameter than wild type (P = 4.2^.^ 10^−7^ by two-tailed t-test) and adopted a more dome-like shape. A dome-shape is typical for the inflorescence meristem. Scanning electron microscopy demonstrated that the *eif3h* mutant apex in a 3-week-old seedling could be significantly enlarged at a time when wild type has begun to produce flowers (**Figure 1C, D and H**). The mutant meristem has enlarged in part through cell proliferation and in part through dramatic cell expansion, a sign that the meristematic cells have adopted a differentiated fate. Cell diameters in our wild-type meristems were 4.2±1.2 micron, close to expectations [28]. For the enlarged meristem in the *eif3h* mutant (**Figure 1D and H**), the cell diameter in the apex was 14.2±4.0 microns. In contrast, the transverse diameter of differentiated petiole cells was similar between wild type and *eif3h*, i.e. 13.3±3.2 micron and 15.8±3.7 micron, respectively. In these mutant plants, the shoot apex eventually formed a large dome-shaped structure visible to the naked eye (**Figure 1F** **,** compare wild type in **1E**). However, this phenotype was not fully penetrant, as many *eif3h* plants will produce normal-sized inflorescence meristems (**Figure 1N, O**), in contrast to the *clv3* mutant (**Figure 1P**). Plants that formed such a dome-shaped apex typically senesced and died without initiating any additional leaves (**Figure 1G**). Occasionally, plants that had suspended leaf formation would eventually initiate multiple new shoot apices late in development (3 out of 78, 4%) (**Figure 1I**). Radialized leaves were common on the *eif3h* apex **(** **Figure 1J–M**, **Table 1** **).** The majority of these leaves were abaxialized as judged by the lack of trichomes at the juvenile stage. An enlarged meristem is characteristic of mutations in the repressors of stem cell proliferation, *CLAVATA1* and *CLAVATA3*. Like *clv1* and *clv3* mutants, the *eif3h* mutant occasionally (10–20%) produced fruits with more than the regular two carpels (**Figure S1A, B**), bifurcated (**Figure S1C**) or fasciated stems (**Figure S1D**). Other typical abnormalities in *eif3h* are shown in **Figure S1E–G**). In summary, the vegetative phenotypes observed in *eif3h* mutant plants suggest expansion of SAM size accompanied by a failure to initiate new organs. In plants that managed to flower, SAM enlargement was less pronounced, but could still be deduced from abnormalities, such as fasciated stems, abnormal organ positioning and an enlarged gynoecium.

**Table 1:** Meristem abnormalities in the *eif3h* mutant.

	*eif3h*	Wild type
Meristem diameter1)	93±14 µm	71±10 µm
Meristem morphology2)		
Openly visible and enlarged	29/49	0/50
with radialized leaves	17/29	none
Not openly visible	20/49	50/50
with radialized leaves	8/20	0/50

1) Determined in situ by differential interference microscopy from 22 (*eif3h*) and 52 (wild type) 12 day old seedlings.

2) Determined as in **Figure 1J** under a stereomicroscope.

### eIF3h Boosts Translation of *CLV1* mRNAs

**Figure 2:**
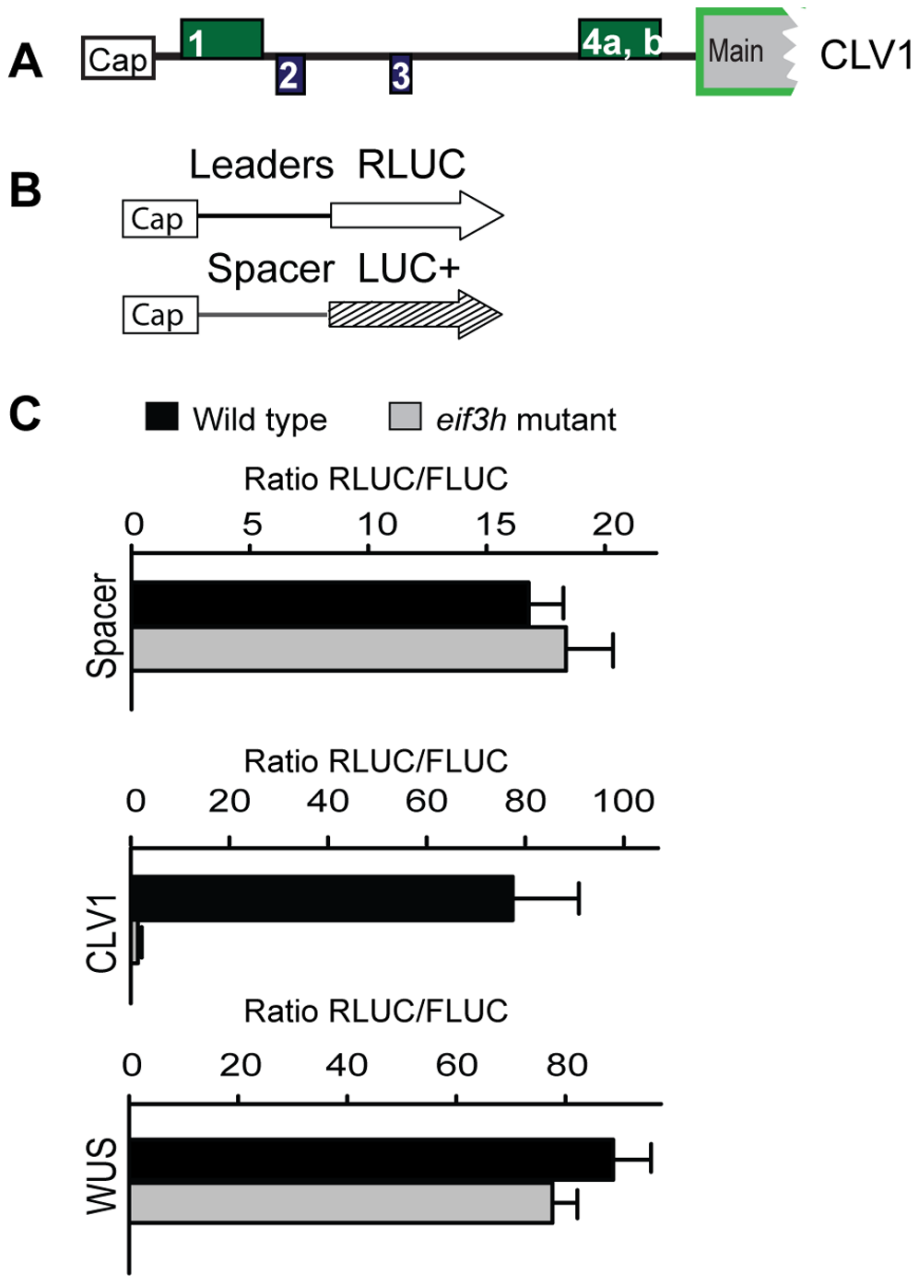
The 5′ leader of the CLV1 mRNA renders translation dependent on eIF3h. (**A**) The 5′ leader of *CLV1* harbors multiple uORFs. The boxes stand for uORFs that are in the –1 frame (green), or in the +1 frame (blue) with the main ORF. The cDNA sequence corresponds to the longest known gene model: *CLV1* (At1g75820.1). (**B**) Schematic view of the mRNAs for protoplast transformation. mRNAs were prepared by *in vitro* transcription with SP6 RNA polymerase. An equal amount of internal control (Spacer-LUC+) mRNA was added to the 5′ leader-RLUC mRNA to be tested as an internal control for transformation efficiency. (**C**) Translational efficiency on the *CLV1* and *WUS* 5′ leader is expressed as the mean RLUC/FLUC ratio with standard errors from three replicate transformations.

eIF3h counteracts the translational repression by uORFs [13]–[15]. Among genes involved in SAM maintenance, the receptor kinase *CLV1* harbors five uAUGs, suggesting that *CLV1* is a potential client of eIF3h. A protoplast transformation assay based on *in vitro* transcribed mRNA was adopted to observe the translation efficiency of specific mRNA 5′ leaders in the *eif3h* mutant. The translational efficiency on the uORF-containing *CLV1* leader was lower in *eif3h* than wild type, whereas the translation on the uORF-free *WUS* leader was not altered **(** **Figure 2B, C** **)**. The ribosomal occupancy of the native Arabidopsis *CLV1* mRNAs was also reduced in the *eif3h* mutant compared to wild-type (**Table 2**).

**Table 2:** Polysome loading state of selected mRNAs in *eif3h* mutant versus wild type.

Gene	AGI	TL *eif3h*/WT	uORFs
*CLV1*	At1g75820	−0.89	4
*AS1*	At2g37630	−1.44	3
*TOADSTOOL*	At3g02130	−1.65	NA
*CORYNE*	At5g13290	−0.36	4
*CLV2*	At1g65380	−0.46	0
*CORONA*	At1g52150	−0.59	8
*SPLAYED*	At2g28290	−0.20	2
*KNAT1/BP*	At4g08150	−0.40	2
*ARF4*	At5g60450	−0.59	3
*ARF5*	At1g19850	−0.48	6
*ARF6*	At1g30330	−0.20	6
*ARF7*	At5g20730	−0.61	4
*ARF11*	At2g46530	−0.47	6
*ARF18*	At3g61830	−0.76	2
*HY5*	At5g11260	+0.17	1
*ATBZIP11*	At4g34590	−0.65	4
*TIR1*	At3g62980	+0.18	1
*AUX1*	At2g38120	+0.31	1

Note: TL *eif3h*/WT. Translation state (TL) is the ratio of mRNA in polysomal and non-polysomal RNA fractions; it is log-transformed and unitless. When compared between *eif3h* mutant and wild-type seedlings, negative numbers indicate that the mRNA is less polysomal, i.e. undertranslated in the *eif3h* mutant. The values are means from duplicate polysome microarrays [13]. TOADSTOOL and the heterodimer of CLV2 and CORYNE are receptor like kinases that can function as CLV3 receptors in parallel to CLV1. Data for *ARFs*, *TIR1* and *AUX1* are included for the purpose of calibration, given that *ARFs* are also undertranslated in *eif3h* while *TIR1* and *AUX1* are not [16]. The median value from 8832 genes was −0.10±0.21. *TOADSTOOL* and *AS1* and *CLV1* rank 19th and 55th and 495th, respectively, among cytosolic mRNAs. uORF numbers are from TAIR10. NA, not available.

**Figure 3:**
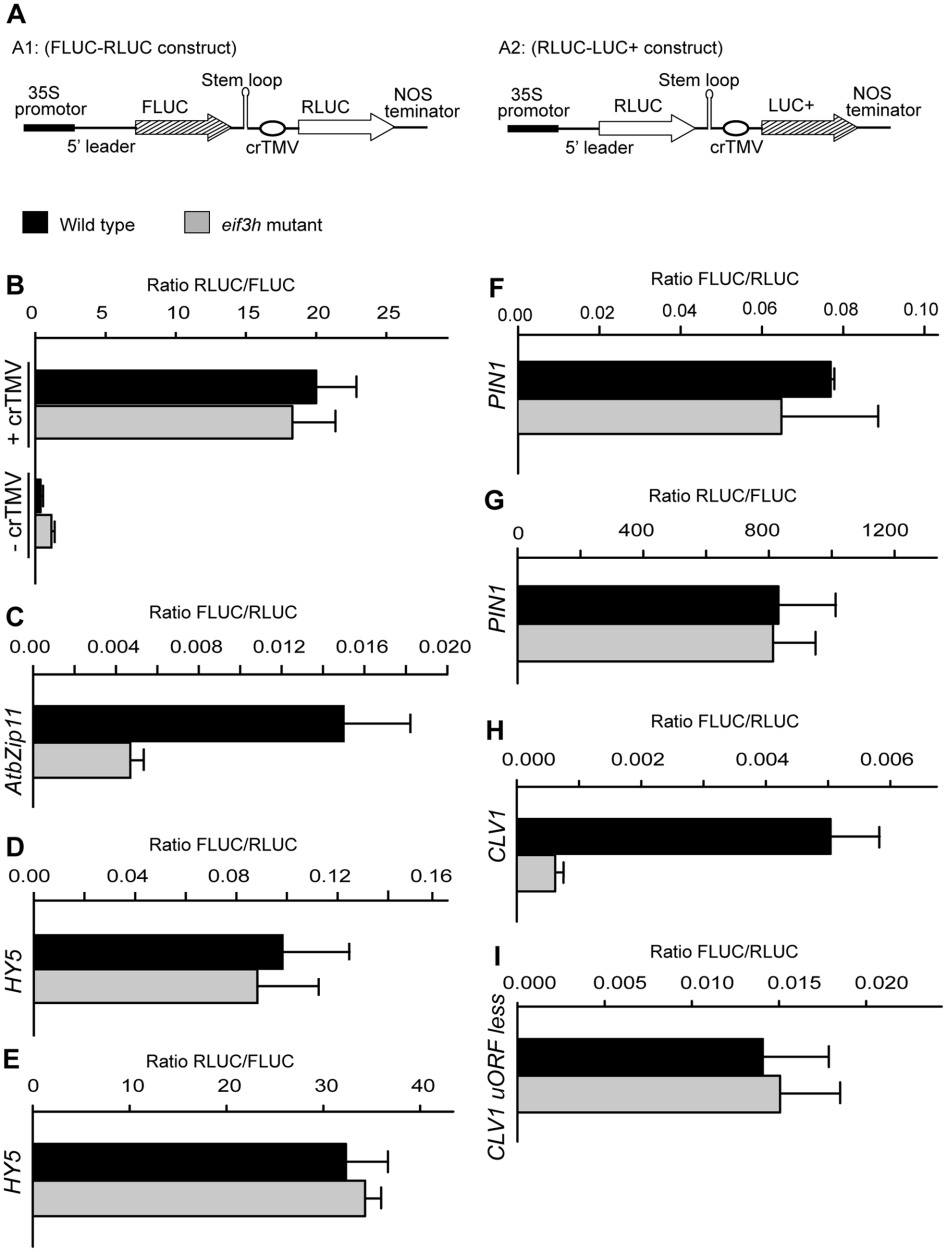
Translation assays with reference gene driven by the crTMV intergenic sequence. (**A**) Structure of the expression plasmids. In A1, the experimental FLUC reporter gene is transcribed by the 35S promoter and harbors the 5′ leader to be tested for translational efficiency. The RLUC reference gene is located further downstream on the same plasmid and is expressed due to transcriptional promoter activity of the crTMV sequence element. In A2, the experimental reporter is RLUC and the reference is LUC+. (**B–I**) Plasmids were transiently transformed into 10 day old wild-type or *eif3h-1* seedlings. The expression is given as the ratio of the reporter luciferase activity divided by the reference luciferase from three replicate transformations with standard error. (**B**) The A1 construct with or without the crTMV sequence was used to confirm that the crTMV element is not eIF3h dependent. In this exceptional case, data are shown as downstream : upstream activity. (**C–H**) Tests of four plant 5′ leaders. (**C**) *AtbZip11;* (**D**) *HY5*; (**E**) *HY5* leader in the RLUC-LUC+ construct (A2). *AtbZip11* and *HY5* leaders served to evaluate the translation assay system. (**F, G**) *PIN1*; (**H**) *CLV1;* (**I**) *CLV1* uORF-less. *HY5* has only one very short uORF and *PIN1* has none.

The translation efficiency under the control of the *CLV1* 5′ leader was further examined using a novel DNA-based, single-plasmid, assay, in which the translational reporter (upstream) and the transformation control (downstream) were fused via a sequence from the crucifer strain of tobacco mosaic virus (crTMV), which was reported to act as an internal ribosome entry site [29] (IRES; **Figure 3A**). The crTMV element had minimal IRES activity in our hands, but had fortuitous promoter activity when the plasmids were transformed into Arabidopsis protoplasts (**Figure S2**). However, since the expression from the crTMV element was not affected by the *eif3h* mutation (**Figure 3B**), the constructs were deemed adequate for determining the translation potential of specific mRNA leader sequences. For additional validation, we confirmed that the leader of *AtbZip11* was *eif3h-*dependent in this assay, while *HY5* was not, as expected [14] (**Figure 3C–E**). The uORF-less *PIN1* leader was also not affected by the *eif3h* mutation (**Figure 3F, G**). However, the *CLV1* leader again showed clear eIF3h-dependence (**Figure 3H**).

### Multiple uORFs in the *CLV1* Leader Contribute to its eIF3h Dependence

**Figure 4:**
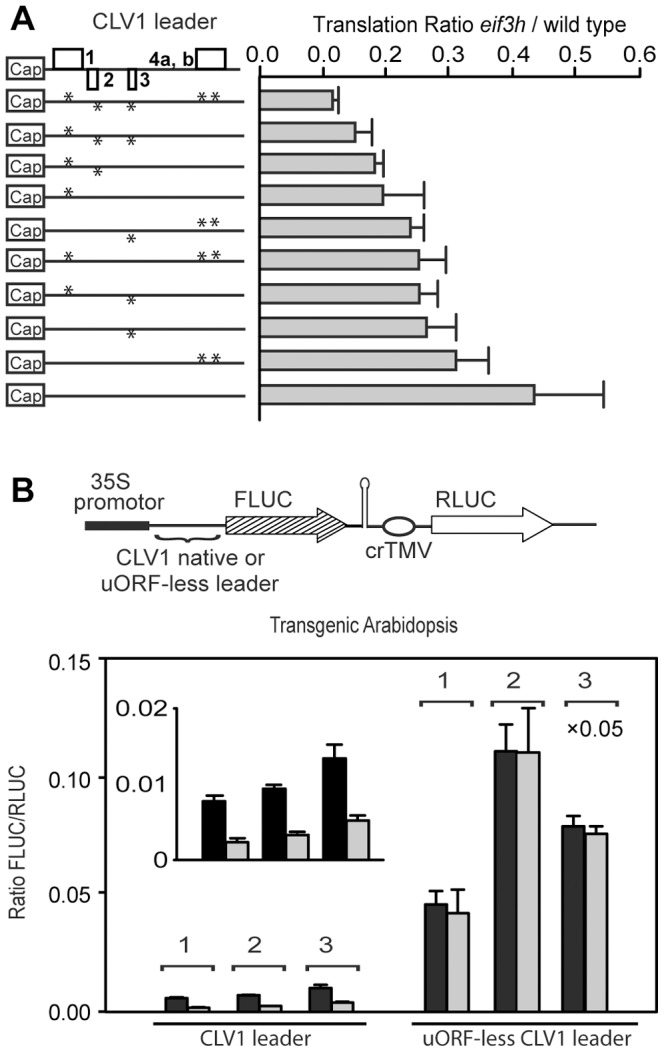
Removal of uORFs from the *CLV1 5′* leader reduces its eIF3h dependence. (**A**) Transient dual luciferase assays were performed in protoplasts as in **Figure 2** but data are presented as the relative expression in the *eif3h* mutant as compared to wild type with standard deviations. Asterisks represent up to five uAUGs and open boxes represent uORFs. (**B**) Stable transgenic plants harboring the dual-luciferase construct illustrated at the top. The CLV1 native leader or uORF-less leader is linked to FLUC, and the RLUC ORF driven by the crTMV-element serves as a reference. Translational efficiency of the 5′ leader was established via dual luciferase assays in 10-day-old seedlings from three independent transgenic lines. Statistical significance was determined by t-test (***for p-value <0.001 and **for P-value<0.05). x0.05: FLUC activities of one particularly highly expressing line were multiplied by 0.05 for convenience of display.

The four uORFs present in the *CLV1* 5′ leader have 16, four, one, and twelve codons respectively; the fourth one contains an internal AUG codon. Translation assays using in vitro transcribed mRNAs indicated that the dependence on eIF3h was significantly reduced when all five uAUGs were removed (**Figure 4A**). uORFs1 and 2 contributed most strongly to eIF3h dependence (3^rd^ and 4^th^ constructs), while uORF3 or uORF4 alone (5^th^, 8^th^ and 9^th^) had less of an effect. uORFs 1 and 2 also caused the largest absolute reduction in FLUC activity (not shown). We note that, even on the uORF-less *CLV1* leader, expression was lower in *eif3h* compared to wild type (**Figure 4A**). Among other possible reasons, this might be due to the length of the mRNA, which is a factor in its ribosome occupancy in *eif3h* [13], or there might be fortuitous initiation at non-AUG codons in the 5′ UTR. Nonetheless, eIF3h mitigates the cumulative inhibition of translation caused by multiple uORFs of different length and position.

The eIF3h dependence of translation on the native and uORF-less *CLV1* leaders was further examined after stable transformation of *eif3h*-heterozygous plants. Dual-luciferase assays were performed after Mendelian segregation of wild-type and *eif3h* mutant seedlings in the progeny (**Figure 4B**). The uORFs clearly attenuated translation in this assay. While the native *CLV1* leader yielded less expression in the *eif3h* mutant than wild type for all three transgenic lines tested, the uORF-less *CLV1* leader drove equal expression levels in both genotypes (**Figure 4B**). The reduced FLUC expression from the *CLV1* mRNA in *eif3h* could not be attributed to reduced transcript stability (**Figure S3**). Notwithstanding the quantitative disparity in the eIF3h defect between transient and stable expression, one may conclude that the uORF-studded *CLV1* leader requires eIF3h for maximal expression under both conditions.

### *WUS* and *CLV3* Gene Expression in the *eif3h* Mutant

**Figure 5:**
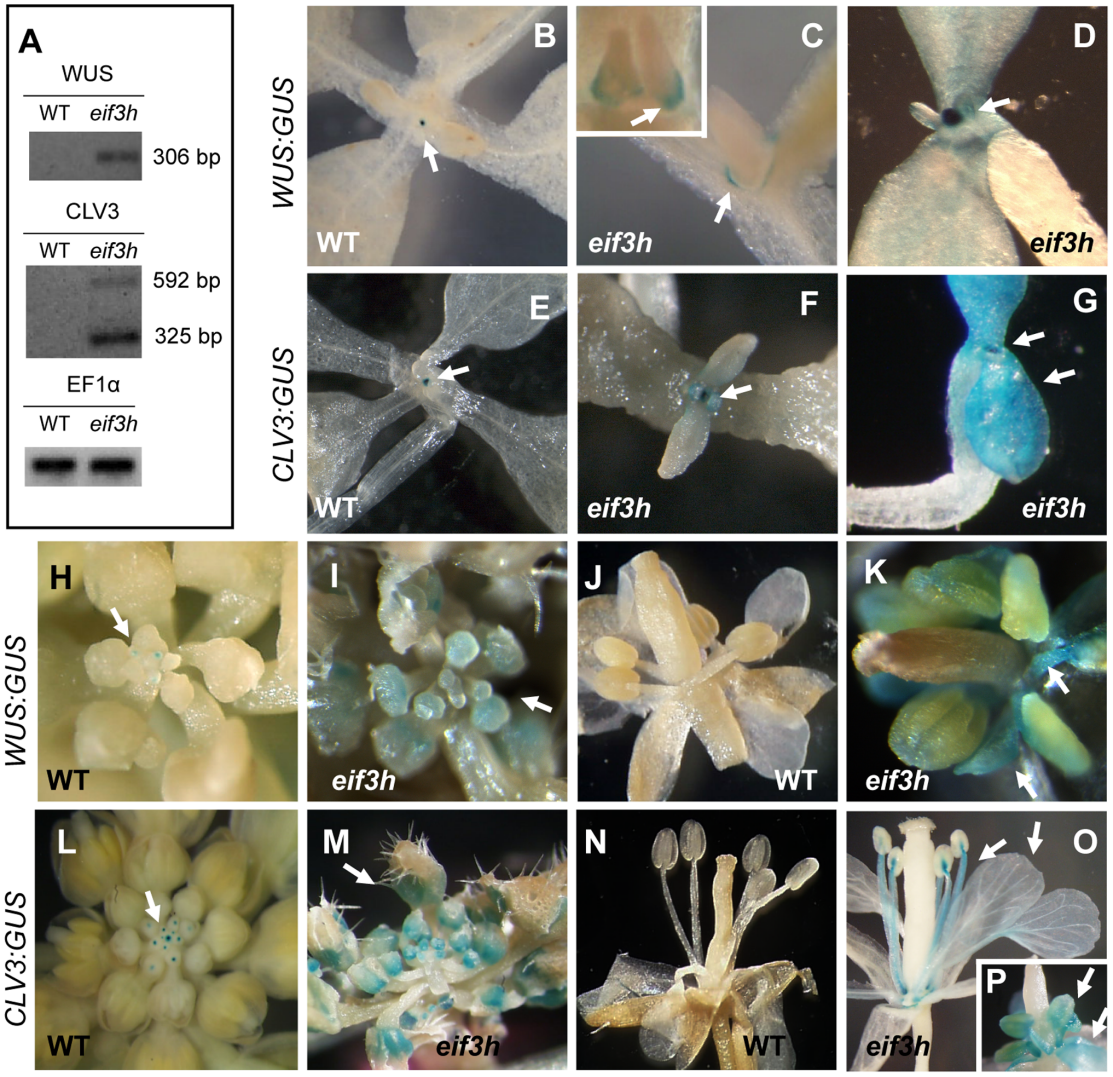
*WUS* and *CLV3* expression in the *eif3h* mutant. (**A**) Reverse transcription (RT) PCR results for *WUS* and *CLV3* mRNAs from 2 week old plants along with translation elongation factor 1α as a control. In *eif3h*, two *CLV3* transcripts corresponding to gene models At2g27250.1 and At2g27250.3 were detected. To detect the transcripts in wild type, more amplification cycles are needed. (**B–D**) *WUS:GUS* expression [57] in wild type (**B**) and *eif3h* (**C, D**) seedlings. (**E–G**) *CLV3:GUS* expression in wild type (**E**) and *eif3h* (**F, G**) seedlings. (**E**) is at half the magnification of F and G. (**H, I**) *WUS:GUS* expression in wild type (**H**) and *eif3h* (**I**) inflorescences. (**J, K**) *WUS:GUS* expression in wild type (**J**) and *eif3h* (**K**) flowers. (**L, M**) *CLV3:GUS* expression in the wild type (**L**) and *eif3h* (**M**) inflorescences. (**N, O**) *CLV3:GUS* expression in wild type (**N**) and *eif3h* (**O**) flowers. (**P**) shows elevated *CLV3:GUS* expression in stamens and petals of a developing *eif3h* flower bud.

Because *CLV1* suppresses expression of the stem cell regulator *WUSCHEL*, any reduction in translation of CLV1 in the *eif3h* mutant would be expected to increase *WUS* transcription, which will in turn promote *CLV3* transcription according to the canonical CLV-WUS feedback regulation. Indeed, RT-PCR results indicated that *WUS* mRNA and two *CLV3* mRNAs were overexpressed in the *eif3h* mutant (**Figure 5A**). Moreover, while expression of *WUS:GUS* and *CLV3:GUS* promoter:reporter transgenes were restricted to a small domain in the shoot apex of wild-type seedlings (**Figure 5B, E**), in the apex of *eif3h* mutant seedlings they tended to be expressed more highly and also ectopically around the base of young leaves (**Figure 5C, F**) and in the cotyledons (**Figure 5D, G**). In keeping with the incomplete penetrance of the meristem overgrowth defect in *eif3h-1*, the size of the meristematic expression domain was still near normal in this experiment. *WUS:GUS* expression was also elevated in the inflorescence tip in the *eif3h* mutant (**Figure 5H–I**), and could often be seen ectopically in floral organs, such as stamens, petals (**Figure 5J–K**), and ovules (data not shown). Consistent with elevated *WUS* expression, the *CLV3:GUS* was also expressed more highly (**Figure 5L–M**) in the *eif3h* inflorescence and ectopically in floral organs (**Figure 5N–P**). Although ectopic *CLV3:GUS* was observed in the *eif3h* mutant embryo, its level in the embryonic SAM was in the normal range (data not shown). Together, these results are consistent with the notion that translational defects in the *eif3h* mutant cause activation of *WUS*, which in turn contributes to the meristem expansion observed in the *eif3h* mutant.

### Relationship between eIF3h and ASYMMETRIC LEAVES1

**Figure 6:**
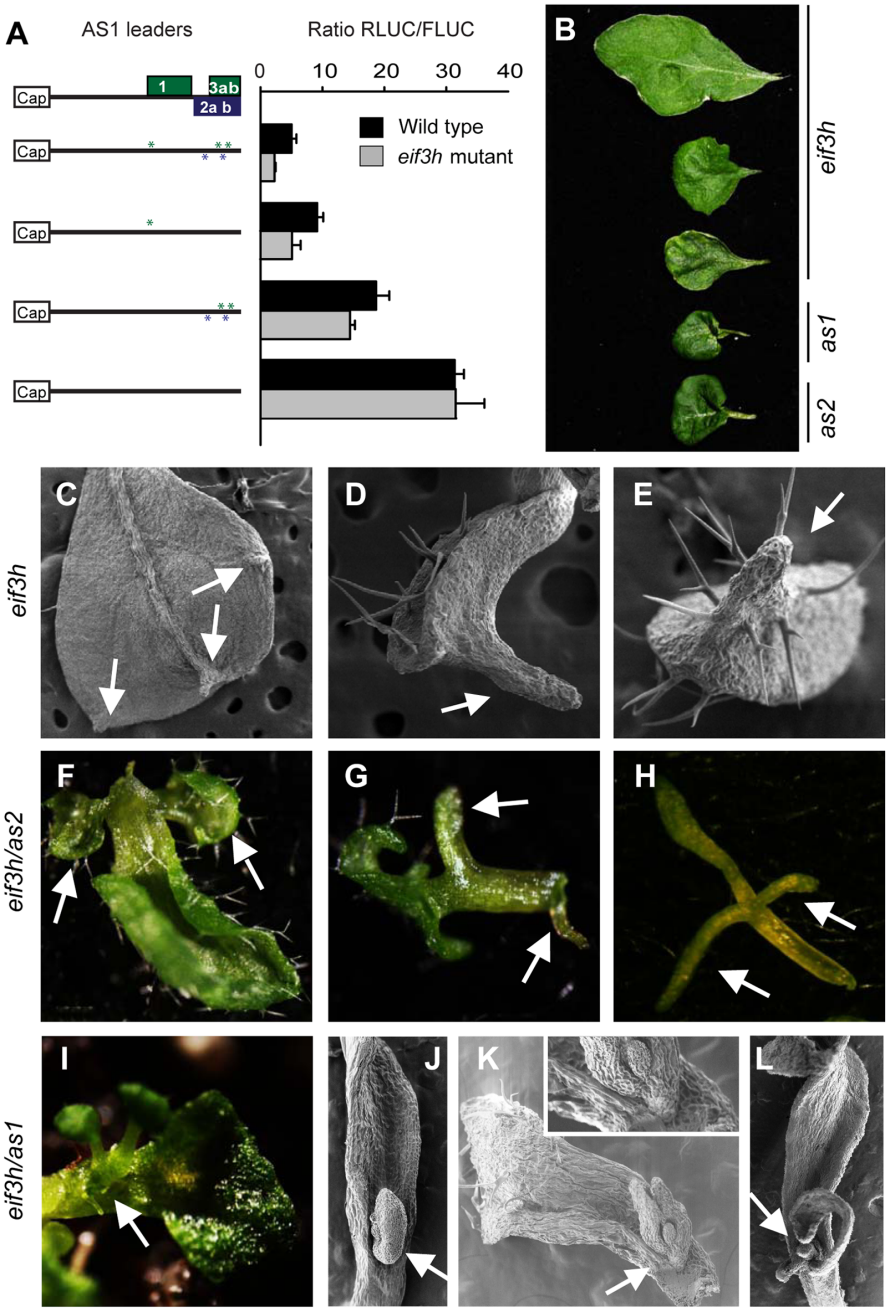
Reduced translation behind the *ASYMMETRIC LEAVES 1* leader in the *eif3h* mutant, and defects on leaf polarity in the *eif3h* mutant and *eif3h/as2* or *eif3h/as1* double mutants. (**A**) Translation assay results for the *AS1* (At2g37630.1) leader and its uORF-removed variants. uAUGs in the *AS1* leader were mutated as described in material and methods for removing uAUGs from the *CLV1* leader. The translation assay was performed as in **Figure 2** and data analyzed as in **Figure 4**. Statistical significance was determined by t-test (**for p-value <0.05). (**B**) Rosette leaves of *eif3h*, *as1* and *as2* (At1g65620) mutants. (**C–E**) Scanning electron microscopic images for *eif3h* rosette leaves with outgrowth on the abaxial side. Arrows point to outgrowths without (**C** and **D**) or with (**E**) trichomes. (**F–G**) Rosette leaves and needle-like leaf of the *eif3h/as2* double mutant with additional leaflets. Arrows point to an expanded leaflet (**F**), needle-like leaflets (**G**) and needle-like leaflets on a needle-like leaf. (**I–L**) Ectopic structures growing on the *eif3h/as1* double mutant rosette leaves. Arrows point to an ectopic shoot growing (**I**) and SEM images of ectopic ovule like structures (**J, K, L**).

The radialized leaves that are often seen in *eif3h* mutant plants toward the end of the growth period may be due to defects in leaf polarity. *ASYMMETRIC LEAVES1* (*AS1*) and *AS2* code for transcription factors that cooperate as adaxializing factors in leaf polarity. In snapdragon, a mutation in the *AS1* homolog, *PHANTASTICA*, alone causes radialized and abaxialized leaves [30], [31], and *AS1/PHAN* possesses a cluster of evolutionarily conserved uORFs [32]. *eif3h* mutant leaves were crinkly, similar to those of *as1* and *as2* mutants (**Figure 6B**). The *AS1* uORFs were indeed inhibitory to translation and caused a slight dependence on eIF3h (**Figure 6A**). In addition, the *AS1* mRNA has reduced ribosome occupancy in the *eif3h* mutant (**Table 2**). Together, these results suggest that crinkly and radialized leaves in *eif3h* may be due in part to poor translation of AS1.

The *eif3h* mutant occasionally had ectopic outgrowths on its leaves **(** **Figure 6C–E** **)**, but these did not normally reveal any pluripotential stem cell character. However, the *eif3h* leaf phenotype was strongly enhanced by both *as1* and *as2* mutations to similar degrees. Double mutants formed elaborate outgrowths on their leaves, which suggested that the leaf tissue can adopt meristematic potential (**Figure 6F–L**). The enhancement of the *as2* phenotype in particular suggests that eIF3h may be rate limiting for *AS1* expression.

## Discussion

Maintenance of the stem cell population in the SAM is critically important for the continuous initiation of lateral organs including leaves, branches, and flowers. The underlying regulatory feedback loop composed of the *WUS* and *CLV* genes has been studied intensively from different angles, including its interface with auxin and cytokinin [18], [33], [34]. This study revealed another previously underappreciated aspect of SAM maintenance, an interplay between uORF containing mRNAs and the machinery supporting their efficient translation. The striking formation of an enlarged meristematic dome (**Figure 1**) or of a pinformed stem in the *eif3h* mutant [16] suggests that Arabidopsis eIF3h supports stem cell homeostasis, organ initiation, and morphogenesis. We propose that eIF3h does so in part by overcoming the inherent inhibition of translation by clusters of uORFs that reside in mRNAs encoding several regulators of meristem activity.

### Clients of eIF3h Maintain Stem Cell Homeostasis

We identified two new uORF containing clients of eIF3h, *CLV1* and *AS1*, and demonstrated their translational defects in the *eif3h* mutant. The *CLV1* mRNA has reduced polysome loading in the *eif3h* mutant (**Table 2**). The full length *CLV1* mRNA harbors four uORFs in its 5′ leader, which are responsible in part for the eIF3h-dependence of translation of a fused reporter gene (**Figure 2** **,** **3** **,** **4**). Underexpression of *CLV1* should result in overexpression of *WUS* and in turn *CLV3* [19], [26]. *WUS:GUS* and *CLV3:GUS* overexpression was indeed observed (**Figure 5**), along with enlargement of the vegetative SAM (**Figure 1**).

Full length *AS1* mRNA harbors three uORFs. Again, eIF3h maintains ribosome occupancy on the *AS1* mRNA and partially alleviates the translational suppression by its inhibitory uORFs (**Figure 6**). Interestingly, the position of the *AS1* uORFs is highly conserved throughout the dicotyledonous plants, suggesting their regulatory significance [32], although their peptide sequence is not conserved. Underexpression of *AS1* will cause wrinkled leaves, which were observed in the *eif3h* mutant. Underexpression of *AS1* would also sensitize plants to other mutations affecting leaf polarity, such as *as2*, which was also observed. For comparison, the conservation status of uORFs in the 5′UTRs of *CLV1* orthologs will require better cDNA sequence information. However, among nine putative *CLV1* orthologs identified from public genomic DNA sequences of various eudicots (*Vitis*, *Citrus*, three *Brassicaceae*, four legumes), all had upstream AUGs within 120 nucleotides of the main AUG, albeit with a pattern different from Arabidopsis (not shown). In summary, *CLV1* and *AS1* thus join the ranks of several other uORF containing mRNAs that are translated poorly in the *eif3h* mutant, most notably auxin response factors [16], [17].

The phenotypic defects of the *eif3h* mutants are consistent with the simultaneous disruption of multiple translation units, not just *CLV1* and *AS1*. The WUS-CLV circuit is generally robust and not easily perturbed by external circumstances such as nitrogen starvation, or herbicide, conditions that reduce translation. Broader changes, presumably affecting multiple transcripts, will be necessary to disrupt the circuit. The translational attenuation of *CLV1* mRNA in the *eif3h* mutant would certainly not be sufficient to cause the loss of control over the meristem in the *eif3h* mutant plants. Even complete loss of *CLV1* translation should produce only a relatively mild phenotype, i.e. an enlarged SAM and an increased number of flowers initiated on the shoot apex [20], [23]. However, in *eif3h* mutants, the shoot apex often enlarges continuously and stops producing lateral organs, before eventually arresting its growth. Similar enlarged leafless apical domes arise as a consequence of a variety of genetic perturbations, for example upon excessive or ectopic *WUS* expression [35]. Likewise, excessive silencing of Homeodomain-Zipper class III transcription factors, which are adaxial leaf determinants, in conjunction with a mutation of the ERECTA receptor kinase also causes meristem overproliferation [36]. Finally a similar defect arises upon misregulation of the auxin equilibrium in the shoot apex, such as in *pin1 arf5/mp* double mutants, in *yucca aux1* double mutants, as well as in *arf5/mp* mutants treated with the auxin efflux inhibitor 1-*N*-naphthylphthalamic acid [37], [38]. Again, we surmise that it is the mistranslation of multiple regulatory mRNAs that causes similarly severe phenotypes in the *eif3h* mutant.

Notably, *CLV1* functions in concert with several other receptor kinase like genes, *CORYNE*, which also contains uORFs, *TOADSTOOL*, and *CLAVATA2*. Moreover, other genes that regulate *WUS,* such as *CORONA* and *SPLAYED* [39]–[41], harbor multiple uORFs in their 5′ leaders. Not unlike *CLV1*, several of these mRNAs have reduced ribosome occupancy in the *eif3h* mutant, which may well add to the misregulation in the *eif3h* SAM. We suggest that the misregulation of the meristem in the *eif3h* mutant is caused by the combined undertranslation of several if not many meristem regulators. The full range of mRNAs affected by the *eif3h* mutation is unknown, but certainly includes mRNAs with functions beyond meristem maintenance. Aside from translational suppression, some mRNAs are translationally stimulated, for example ribosomal protein mRNAs [13], [16], [17], [42].

A complex system such as the SAM is maintained by multiple positive and negative feedback loops. Induction of *CLV3* by *WUS* maintains the SAM. Besides the well known, stabilizing, negative feedback from CLV3 via the receptor kinases to *WUS*, *WUS* overexpression also causes further repression of *CLV1* expression [43], and overexpression of *CLV3* can downregulate CLV1 protein posttranslationally [44]. If such a system is perturbed simultaneously at multiple steps, as may well be the case when eIF3h activity is defective, the eventual outcome could be of two types. Either the system manages to rebalance itself, or it collapses. The *eif3h* mutant may teeter on this verge, and this may be the reason why only a fraction of *eif3h* mutant plants lose control over the meristem.

While the severe phenotypic defects indicate a major shift away from regular stem cell homeostasis, the *eif3h* mutant evidently retains some ability to translate genes with uORF-containing mRNAs, given that *eif3h* does not consistently phenocopy, for example, severe *arf5/mp* or *arf3/ettin* alleles. To explain this, we invoke that many of the implicated client genes are transcribed from multiple transcription start sites, which may result in shorter 5′ leaders with fewer uORFs [45]. Moreover, one should also expect significant leaky ribosome scanning across those uAUGs that are in a weak initiation context.

### Translational Control in Arabidopsis Development

**Figure 7:**
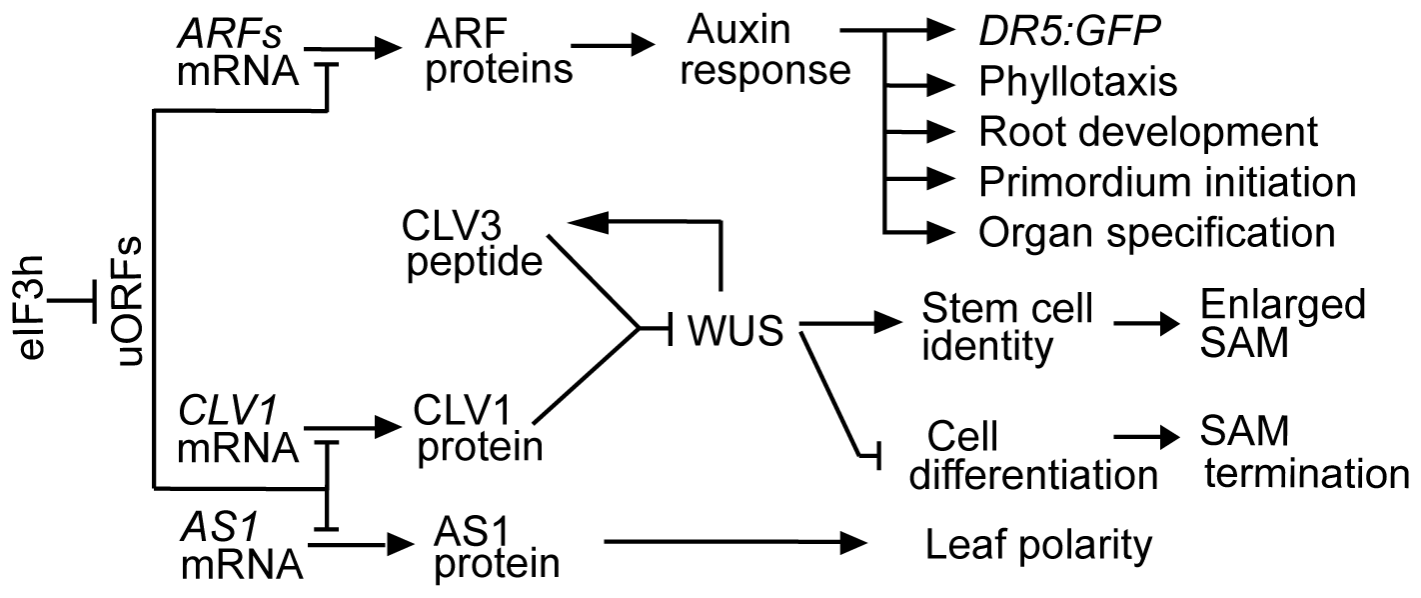
A concept map for the role of eIF3h in *Arabidopsis* SAM maintenance and auxin response. By overcoming the translational repression by uORFs, eIF3h promotes the translation of *ARF*s [16] and *CLV1* and *AS1* (this work), and therefore plays an important role in SAM maintenance and organogenesis.

The concept that translational control of regulatory mRNAs adds a novel layer of gene regulation in the meristem merits further exploration (**Figure 7**). In keeping with this notion, several groups reported that mutations in genes for ribosomal proteins enhance mutations in leaf polarity factors, such as *AS1* and *AS2* [5]–[7]. For example, a double mutant of *rpl4d* and *as2* forms trumpet-like and needle-like leaves [4] similar to those observed in *eif3h as1* double mutants. Certain phenotypes of the *rpl4d* mutant can be partially rescued with specific auxin response factor genes once their uORFs have been removed from their 5′ leaders [46]. Similar to these ribosomal protein mutations, *eif3h* strongly enhanced leaf polarity defects of *as2* and *as1* mutations and often generated radialized leaves on its own. These results indicate that the developmental clients of eIF3h are not restricted to the meristem, but include organogenesis factors. Several ribosomal mutations, including *rpl4d*, cause a pin-like phenotype when combined with *as2* [4]–[7]. A mutation in *rpl4d* can be pin-like when *CLV3* expression is altered, *rpl5a* has pin-like shoots in the Landsberg background [46], and *rpl24b/stv1* enhances *arf3* to form pins [8]. The spectrum of the *eif3h* mutation is clearly reminiscent of these phenotypes seen in ribosomal protein mutants. However, no meristem overgrowth phenotype like the one presented here for *eif3h* has been described for these ribosomal mutants on their own (e.g. see [47]).

We propose that the phenotypic spectrum of the *eif3h* mutant points to a group of mRNAs that are particularly sensitive to defects in the translation apparatus. Evidently, the phenotype of mutants in the translation apparatus will be driven by those mRNAs that have special requirements during translation. mRNAs with uORFs pose special requirements for translation, because, either, the translation machinery must perform two successive initiation events on the same mRNA. Or the translation machinery must bypass uORFs by leaky scanning. In the absence of either reinitiation or leaky scanning, the main open reading frame will be loaded skimpily with ribosomes, which may trigger other forms of posttranscriptional inhibition such as nonsense-mediated decay [48]. Here, we demonstrated, using the *eif3h* mutant as an example, that developmental regulatory genes such as *CLV1* possess uORFs that render them sensitive to mutations in the translation apparatus.

The value of this study probably lies less in laying bare specific new mechanisms in stem cell regulation. Rather, it opens the window onto a previously underappreciated RNA sequence element that is present in a multitude of mRNAs that function in stem cell maintenance. What aspect of translational control in the meristem should be attributed to uORFs? uORFs may fine-tune the expression of many developmental regulator genes. In addition, weak transcriptional activity is believed to be noisy and stochastic in living cells [49], [50]. Because uORFs generally reduce translation they may serve to permit a high, by inference less noisy, transcription rate while keeping the rate of protein synthesis at the low level expected for a regulatory protein. One role of the eIF3h protein appears to be to maintain a moderate efficiency of translation on such uORF containing mRNAs.

## Materials and Methods

### Plant Growth Conditions and Transformation

Growth conditions for wild type (Wassilewskija ecotype) and the *eif3h*-1 allele, which harbors a T-DNA insertion in the 10th of 12 exons and results in a truncated protein, have been described [14]. Transgenes were transformed into heterozygous *eif3h* mutant plants using floral dip of *Agrobacterium*. Transgenic plants were selected on 10 mg/l Basta and verified by PCR.

### Molecular Cloning

The plasmids for in vitro transcription were made in the TA-cloning vector pKRX [51] and contained the SP6 phage promoter, the translational leader from tobacco etch virus (TL) and the coding region of firefly luciferase (FLUC) or LUC+ (from pGL3-basic, Promega, Madison, WI) or Renilla luciferase (RLUC; [52]) followed by a 70 nucleotide long poly-A tail. The TL 5′ leader was replaced with the respective leader sequence to be assayed. The 5′ leader called Spacer is the multiple cloning site of pGL3-basic.

The crTMV based dual-luciferase expression cassettes were assembled in pBluescript II using the cauliflower mosaic virus 35S promoter, tobacco etch virus leader (TL), and RLUC, the crTMV internal ribosome entry site sequence from plasmid pYY376 [29], [53], FLUC or LUC+, and the nopaline synthase terminator. A truncated crTMV element retaining only 7 bp on the left and 20 bp on the right end was generated as a negative control. Stop codons were introduced between the upstream luciferase gene and the IRES with the oligonucleotides (LOOPADP-for: 5′ GATCTATCTAGTCTAGATAGCGTAGCCTAGGGGTG ACCACTAGTACCGGTGACGTCGG 3′ and LOOPADP-rev 5′ CGCGCCGACGTCACCGGT ACTAGTGGTCACCCCTAGGCTACGCTATCTAGACTAGATA-3′. A stem loop (ΔG = −41.7 kcal/mol) was introduced into the SpeI site in the loop adaptors by annealing the two oligos LOOP-for: 5′ CTAGAGCCACCACGGCCCCCAAGCTTGGGCCGTGGTGGCT 3′ and LOOP-rev: 5′ CTAGAGCCACCACGGCCCAAGCTTGGGGGCCGTGGTGGCT 3′ (A1 in Figure 3A) [54].

### Site Directed Mutagenesis

The primers for removing uAUGs from the *CLV1* leader are listed in **Table S1**. Briefly, wild type *CLV1* leader was amplified with primers AT1G75820-FOR1 and REV1, and cloned between the SP6 promoter and RLUC in a pKRX vector. The *CLV1* leader was re-amplified with primers AT1G75820-FOR2, REV1 and cloned between SP6 and RLUC as before to remove the first uAUG. To further remove uAUG 2, two short PCR products made with M13-FOR, AT1G75820-REV3 and AT1G75820 FOR3, RLUC-REV were fused by a double template PCR with primers M13-FOR and RLUC-REV. Using the double template PCR product as template, a similar approach was applied to further remove uORF 3, and 4a, 4b with primers AT1G75820-FOR4, REV4 and primers AT1G75820-FOR5, REV5 respectively. Equivalent procedures were followed for mutagenesis of the *AS1* 5′ leader.

### Microscopy

For scanning electron microscopy plant material was dissected and placed into 0.1M sodium cacodylate buffered 3% glutaraldehyde for 60 minutes. Samples were then washed in buffer over 30 minutes before being post-fixed in buffered 2% osmium tetroxide for 60 minutes. Samples were washed in water, dehydrated in a graded ethanol series then critical point dried in CO_2_. Once dried, samples were mounted, coated with gold in a SPI sputter coater and examined with a Zeiss 1525 scanning electron microscope.

### DNA Based Expression Assay after Transient or Stable Transformation

Wild-type and *eif3h* mutant plants were grown for ten days on MS agar plates with 1% sucrose. Plasmids carrying dual-luciferase constructs were introduced by particle bombardment as previously described [14]. Transformed seedlings were incubated at 22°C in a lighted growth chamber for 8 hours before assaying for luciferase activity. Activities of the experimental luciferase and the reference luciferase were measured in a single protein extract using the Dual Luciferase system (Promega, Madison, WI) in the TD-20/20 luminometer (Turnerdesigns, Sunnyvale, CA). Mean ratios of experimental and reference luciferase from 3 or 4 biological replicates were used to compare the translation efficiency between wild type and *eif3h* mutant.

### Protoplast Preparation and PEG Mediated mRNA Transformation

Protoplasts were prepared from shoots of wild type or mutant 7-day-old Arabidopsis seedlings [55] and were transformed with 200 ng mRNA using the polyethyleneglycol method [56] as described [15]. The protoplasts were incubated in a 24 well plate for 3 hours in the dark at room temperature, then harvested by centrifugation for luciferase assays.

### GUS Staining

Arabidopsis seedlings or inflorescences were prefixed in 90% acetone for 20 min, rinsed briefly in staining buffer without X-Gluc and infiltrated in staining buffer (0.05M phosphate buffer, pH 7.2; 0.2% Triton X-100; 2 mM potassium ferrocyanide and 2 mM potassium ferricyanide; 2 mM X-Gluc) in vacuum for 30 min, followed by incubation at room temperature for 6 hours. After dehydration with an ethanol series (20%, 35% and 50%), tissue was fixed in FAA (50% ethanol, 10% acetic acid and 5% formaldehyde) for 30 min at room temperature, then dehydrated completely with 70%, 85% and 100% ethanol.

### Reverse Transcription PCR

First strand cDNA were synthesized with M-MLV reverse transcriptase (Promega) and oligo (dT) primers using RNAs prepared from 7 day old Arabidopsis seedlings. PCR for *WUS* and *CLV3* was for 40 cycles and for *eEF1α* was 28 cycles. The gene specific primers for each gene are: *WUS* (forward: 5′-CCCAGCTTCAATAACGGGAAT-3′, reverse: 5′-ACCGTGCATAGGGAAGAGAG-3′), *CLV3* (forward: 5′-cacctcgagCACTCAGTCACTTTCTCTCTAA-3, reverse: 5′-TCAAGGGAGCTGAAAGTTGT-3′) and *eEF1α* (forward: 5′-GATGAGACTTTCGTTATGA TCGAC-3′; reverse: 5′-ATTGAAAACCATAATAAAAAGTCTCAGA-3′). To measure RNA stability, 2-week-old transgenic seedlings were transferred to incubation buffer (1 mM Pipes, pH 6.25, 1 mM sodium citrate, 1 mM KCl, 15 mM sucrose) for 30 minutes, followed by addition of transcriptional inhibitor (100 µg/mL cordycepin; Sigma, St. Louis, MO). Samples were harvested at specific time points and analyzed by RT-PCR using primers listed in **Table S1**.

## Supporting Information

Figure S1 ***eif3h*** **inflorescence phenotypes indicative of defects in meristem regulation. (A)** 3-carpel phenotype of *eif3h*. **(B)** Siliques of *clv1-1* and *clv3-2* showing 3 to 5 carpels are shown for comparison. **(C)** The *eif3h* inflorescence may be bifurcated. **(D)** *eif3h* inflorescence with fasciated shoots. **(E)** *eif3h* lateral branches that are not subtended by cauline leaves. **(F)** *eif3h* inflorescence showing spontaneous arrest of internode elongation in the main apex (arrows) that fails to exert apical dominance. **(G)** Reactivated *eif3h* inflorescence after premature termination.
(TIF)

Figure S2 **The crTMV IRES element has promoter activity.** DNA fragments harboring the elements outlined on the left were isolated from plasmids by gel purification and transformed into tobacco or Arabidopsis seedlings using the particle gun. Luciferase activity was measured in triplicate using a dual luciferase assay (Promega). Means and standard deviations are shown. Orange bar, 35S promoter. Black bar, SP6 promoter. Green arrow, FLUC coding sequence. Blue arrow, RLUC coding sequence. Three red circles, stop codons in all three reading frames. Stem-loop, Forms hairpin-loop when single stranded. Red oval, crTMV IRES element. Hatched red oval, truncated crTMV element.
(TIF)

Figure S3 **Transcript stability of transcripts harboring the** ***CLV1*** **5′ leader.** The decay of mRNA levels for transgenic FLUC and RLUC mRNAs as well as for the highly stable, endogenous, translation elongation factor 1 alpha (EF1α) mRNA was monitored by RT-PCR after blocking transcription with cordycepin. Control amplifications with higher PCR cycle numbers (sat.) were performed for representative samples to confirm that the experimental amplifications had not reached saturation. RNA was isolated from transgenic seedlings used in **Figure 4**. The gene expression cassettes are CLV1-FLUC transgenes used in **Figure 3H** and **3I**.
(TIF)

Table S1 Primers for removing uAUGs from the *CLV1* leader and for RT-PCR.
(DOC)
