# Supplementary figures and images for: Translational Control of Arabidopsis Meristem Stability and Organogenesis by the Eukaryotic Translation Factor eIF3h
Source: PLoS One. 2014 Apr 15;9(4):e95396. doi: 10.1371/journal.pone.0095396 (PMC3988188; doi:10.1371/journal.pone.0095396)

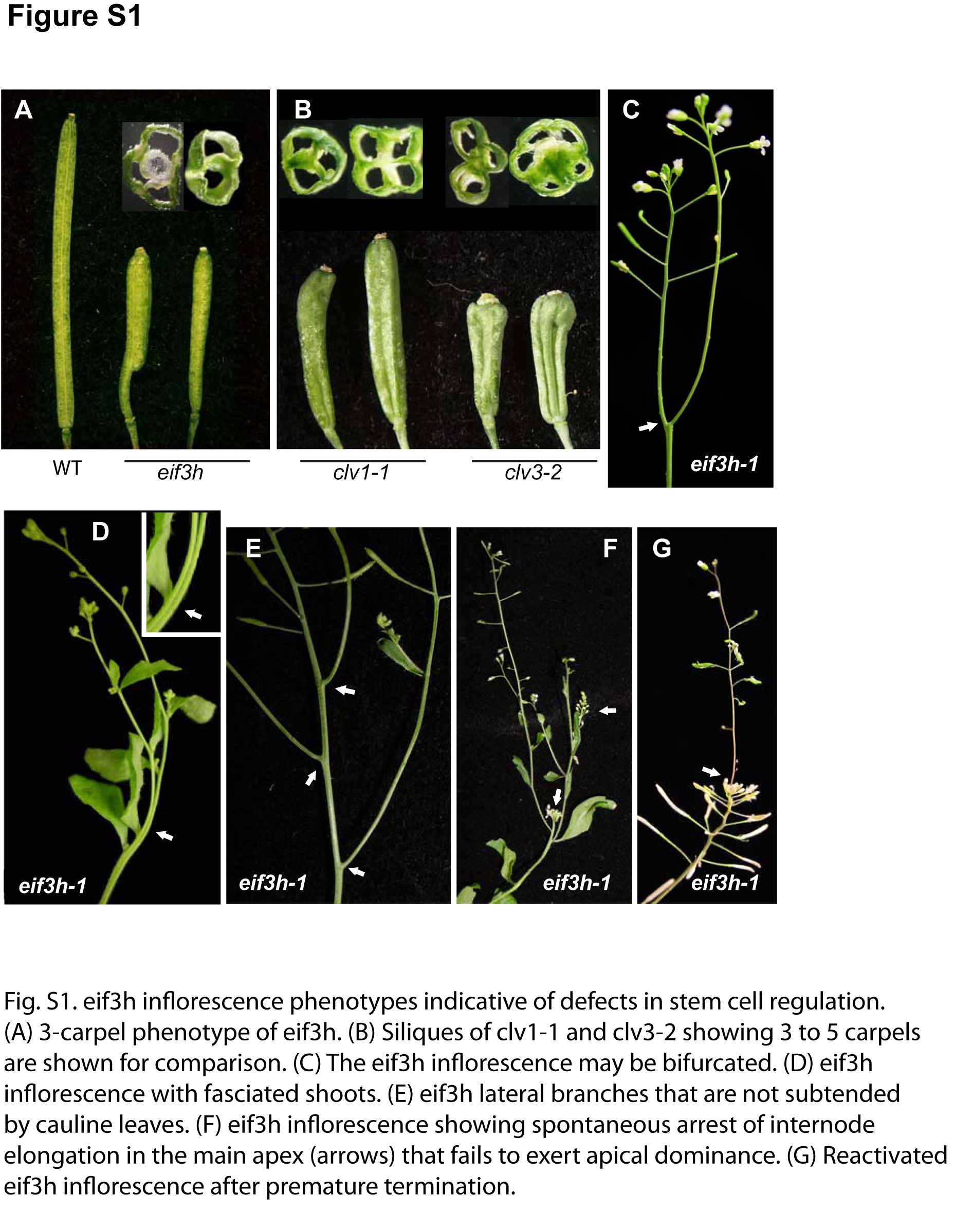

Supplement: Figure S1 — eif3h inflorescence phenotypes indicative of defects in meristem regulation. (A) 3-carpel phenotype of eif3h. (B) Siliques of clv1-1 and clv3-2 showing 3 to 5 carpels are shown for comparison. (C) The eif3h inflorescence may be bifurcated. (D) eif3h inflorescence with fasciated shoots. (E) eif3h lateral branches that are not subtended by cauline leaves. (F) eif3h inflorescence showing spontaneous arrest of internode elongation in the main apex (arrows) that fails to exert apical dominance. (G) Reactivated eif3h inflorescence after premature termination. (TIF) [file pone.0095396.s001.tif]

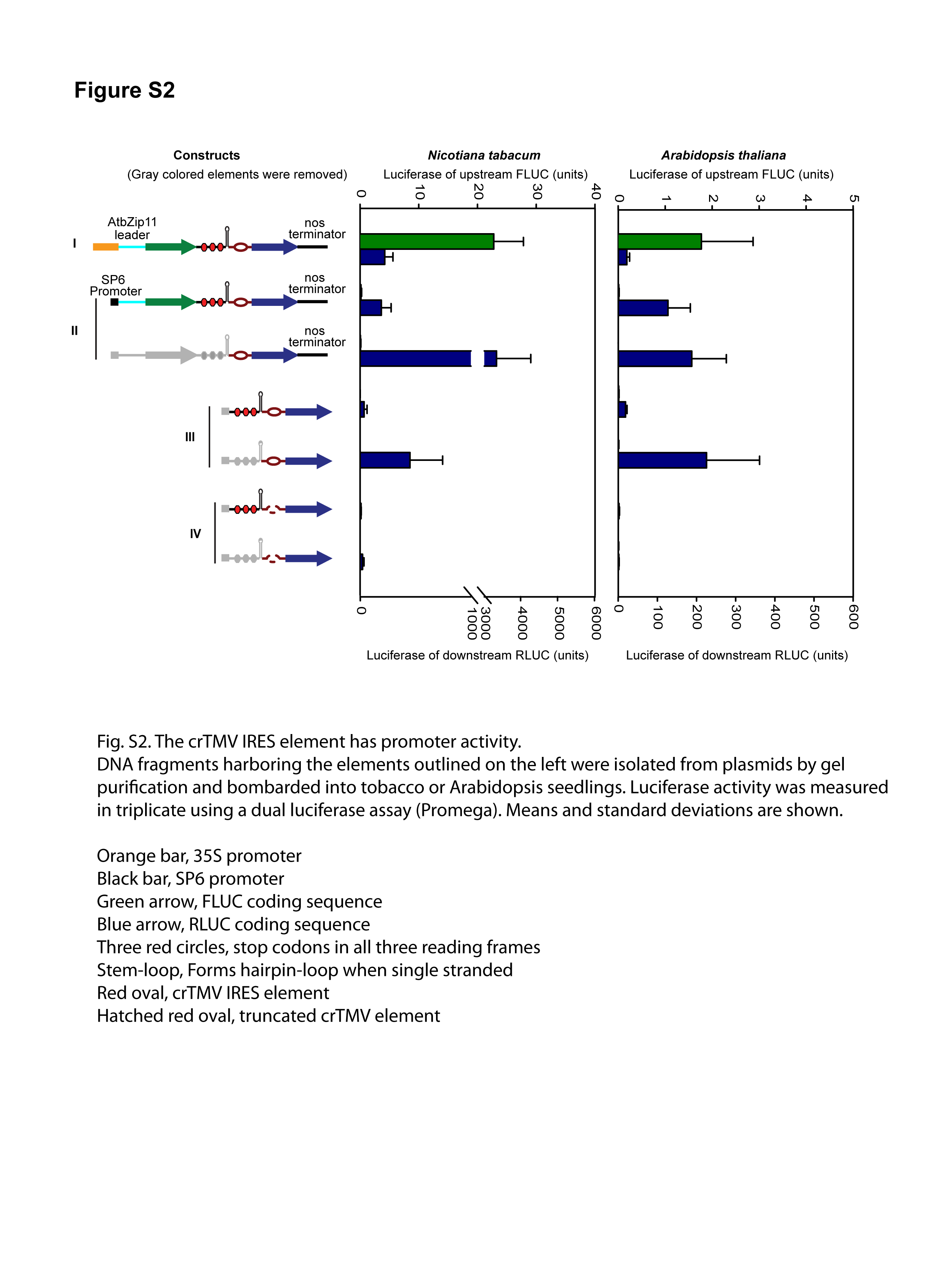

Supplement: Figure S2 — The crTMV IRES element has promoter activity. DNA fragments harboring the elements outlined on the left were isolated from plasmids by gel purification and transformed into tobacco or Arabidopsis seedlings using the particle gun. Luciferase activity was measured in triplicate using a dual luciferase assay (Promega). Means and standard deviations are shown. Orange bar, 35S promoter. Black bar, SP6 promoter. Green arrow, FLUC coding sequence. Blue arrow, RLUC coding sequence. Three red circles, stop codons in all three reading frames. Stem-loop, Forms hairpin-loop when single stranded. Red oval, crTMV IRES element. Hatched red oval, truncated crTMV element. (TIF) [file pone.0095396.s002.tif]

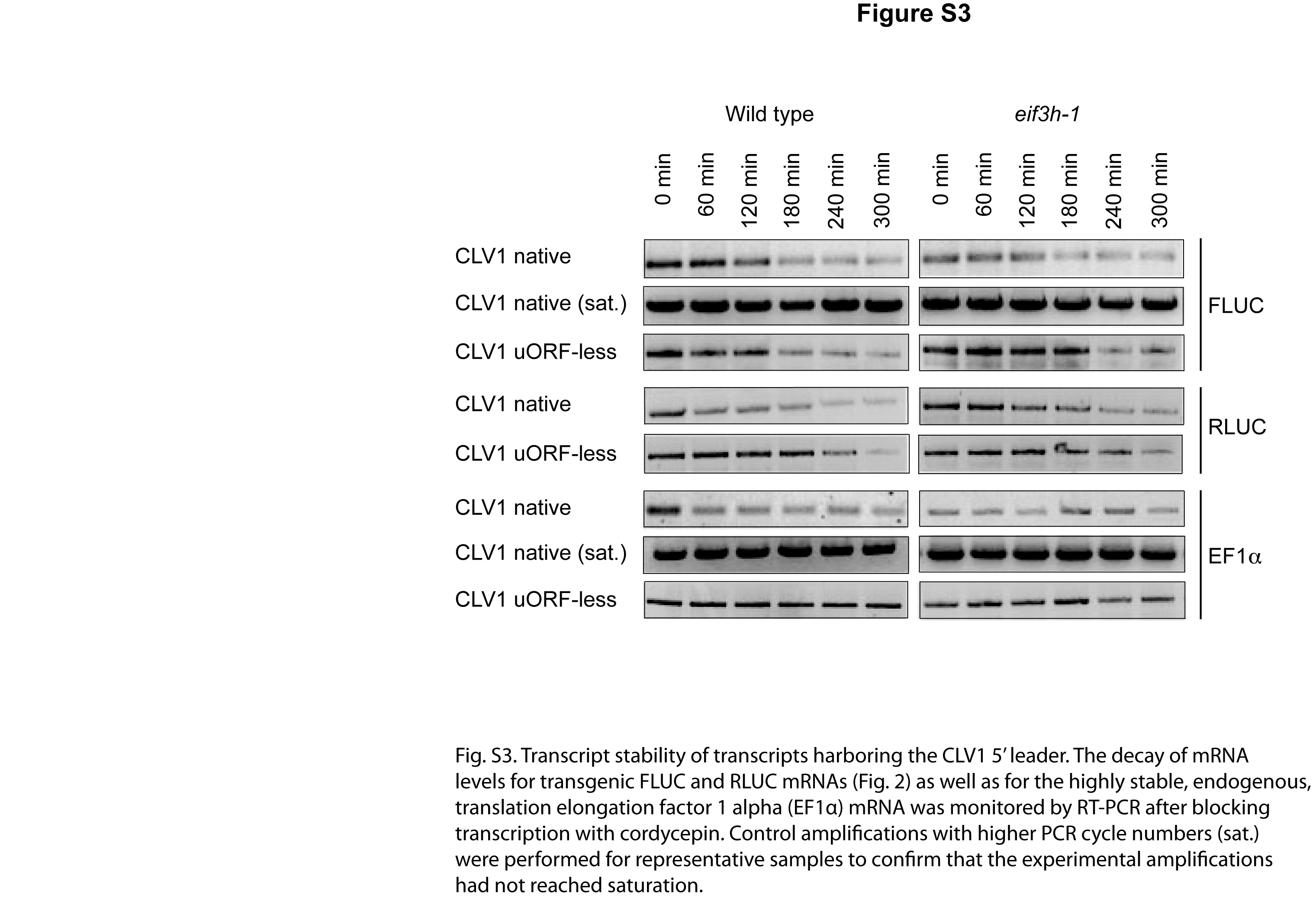

Supplement: Figure S3 — Transcript stability of transcripts harboring the CLV1 5′ leader. The decay of mRNA levels for transgenic FLUC and RLUC mRNAs as well as for the highly stable, endogenous, translation elongation factor 1 alpha (EF1α) mRNA was monitored by RT-PCR after blocking transcription with cordycepin. Control amplifications with higher PCR cycle numbers (sat.) were performed for representative samples to confirm that the experimental amplifications had not reached saturation. RNA was isolated from transgenic seedlings used in Figure 4 . The gene expression cassettes are CLV1-FLUC transgenes used in Figure 3H and 3I . (TIF) [file pone.0095396.s003.tif]
