# Supplementary material for: Translational Control of Arabidopsis Meristem Stability and Organogenesis by the Eukaryotic Translation Factor eIF3h
Source: PLoS One. 2014 Apr 15;9(4):e95396. doi: 10.1371/journal.pone.0095396 (PMC3988188; doi:10.1371/journal.pone.0095396)
Supplement: Table S1 — Primers for removing uAUGs from the CLV1 leader and for RT-PCR. (DOC) [file pone.0095396.s004.doc]

**Supplemental Table T1.** Primers for removing uAUGs in the *CLV1* leader and for RT-PCR.

| **Primer name** | **Primer sequence** |
| --- | --- |
| RLUC-REV | TTGCACCTGGCCCACCACTGG |
| M13-FOR | GTAAAACGACGGCCAGT |
| AT1G75820-FOR1 | CCCctcgagGTAAAATTTCTCTATTCACAAATG |
| AT1G75820-REV1 | CCCccatggTTTTTTAGTGTCCTCTCAGTGAG |
| AT1G75820-FOR2 | GGGctcgagGTAAAATTTCTCTATTCACAAATCAT |
| AT1G75820-FOR3 | TGAAAAATCAACACATAAAAGTATTTTGGCG |
| AT1G75820-REV3 | CGCCAAAATACTTTTATGTGTTGATTTTTCA |
| AT1G75820-FOR4 | CAATATTTATCTAAAGTCACATAAAAG |
| AT1G75820-REV4 | CTTTTATGTGACTTTAGATAAATATTG |
| AT1G75820-FOR5 | CATAATATCCCATTGTTCATCTCTCTG |
| AT1G75820-REV5 | CAGAGAGATGAACAATGGGATATTATG |
| WUS-FOR | CCCAGCTTCAATAACGGGAAT |
| WUS-REV | ACCGTGCATAGGGAAGAGAG |
| CLV3-FOR | ATGTCCGGTCCAGTTCAACA |
| CLV3-REV | TCAAGGGAGCTGAAAGTTGT |
| FLUC-FOR | TTCCATCTTCCAGGGATACGAC |
| FLUC-REV | AGTCTCGAGACTAGTATTTGGACTTTCCGCC |
| RLUC-FOR | GATCAAAGCAATAGTTCACGCTG |
| RLUC-REV | CATTTTTGAGAACTCGATCAACG |
| EF1α-FOR1 | CAGCTAAGGGTGCCGCC |
| EF1α-REV1 | GTCGATCATAACGAAAGTCTCATC |
| EF1α-FOR2 | GATGAGACTTTCGTTATGATCGAC |
| EF1α-REV2 | ATTGAAAACCATAATAAAAAGTCTCAGA |

Note: The upstream ATGs were mutated to ATCs and are underlined in the forward primers.
